# Production and Evaluation of Chitosan from *Aspergillus Niger* MTCC Strains

**Published:** 2011

**Authors:** Natarajan Kumaresapillai, Riyaz Ameer Basha, Rengarajan Sathish

**Affiliations:** a Department of Pharmaceutical Biotechnology, Ultra College of Pharmacy, Madurai, Tamil Nadu, India.; b Department of Pharmacology, Ultra College of Pharmacy, Madurai, Tamil Nadu, India.

**Keywords:** Chitosan production, Aspergillus niger, Antimicrobial activity, *Aspergillus Niger*

## Abstract

Three different *Aspergillus Niger* MTCC strains (872, 1785 and 2208) were used for the production of chitosan. Multivariate growth mediums in varying incubation periods were analyzed for the production. The produced chitosan was characterized by its physical appearance, moisture content (by means of gravimetric method), percentage of ash and solubility (through the Association of Official Agricultural Chemists (AOAC) methods) and also the degree of deacetylation (with Infrared spectroscopy). The infrared (IR) spectrum of produced fungal chitosan shows strong similarity with the IR spectrum of commercial chitosan. Our experimental results concluded that the maximum yield of chitosan (26.1%) was obtained from *Aspergillus niger* MTCC 2208 grown in supplemented Potato Dextrose Broth (PDB) medium, incubated at 30°C for 120 h in 180 rpm. The antimicrobial activity of produced chitosan was tested against five bacteria by Disc diffusion technique, which confirmed that chitosan have minimal antimicrobial activity.

## Introduction

Chitosan is a cationic polymer derived by deacetylation of chitin obtained from crustaceans. Chitosan is the second most polymers used in industries after cellulose (1). Biodegradable and mucoadhesion properties of chitosan have recently led to increasing the interest in the development of slow-release formulations for gastro-retentive drug delivery (2). Chitosan-based dosage forms of this kind could be useful in relation, *e.g.*, to the administration of antibiotics used for eradication of *Helicobacter pylori *in stomach. The present trend, in industrial applications, however, is toward producing high value products, such as cosmetics, drug carriers, feed additives, semi-permeable membranes, and pharmaceutics. The difference in value between the products and the low-cost polymer is one of the main driving forces pushing studies on new applications of chitosan (1). Biotechnology is currently attempting large-scale production of high-value bio-products like monoclonal antibodies, Immobilization techniques, *etc*. Previously, it was investigated that in *Aspergillus niger *cell wall constituents, chitin comprises of 42% and also researchers confirmed that the chitosan content of fungi depends on fungal strains, mycelial age, cultivation medium and conditions (3). The chitosan quantity also depends on the extraction methods (3). *Rhizopus oryzae *TISTR3189 was found to produce maximum yield of chitosan 138 mg/L dry weight, *i.e. *(14%) (4). Nowadays, scientists are paying their attention to find out a new source of organisms which is freely available and easy to cultivate in production of chitosan for the industrial purpose. This study, aimed the utilization of newer fungal strains, by varying the growth medium and incubation periods to get the maximum yield of biomass and excess production of chitosan.

## Experimental

*Microorganisms*

*Aspergillus niger *MTCC 872, *Aspergillus niger *MTCC 1785 and *Aspergillus niger *MTCC 2208 were obtained from IMTEC, Chandigarh, India. *Salmonella typhi, Salmonella paratyphi-*A*, Escherichia coli, Proteus vulgaris, *and *Pseudomonas aeruginosa *were obtained from Bose lab, Madurai, Tamil Nadu, India. The stock cultures *Aspergillus niger *MTCC 872 and MTCC 2208 of organisms were grown on Czapek Yeast Extract Agar (CYA) slant - CYA medium, incubated in aerobic condition in 25°C for 3 and 5 days respectively, and *Aspergillus niger *MTCC 1785 organisms were grown on Czapek Agar slant, incubated in aerobic condition in 25°C for 4 days.

*Medium and culture conditions*

Potato Dextrose Agar (PDA) slant media were used for the cultivation of *Aspergillus niger*. The *Aspergillus niger *MTCC 872, *Aspergillus niger *MTCC 1785 and *Aspergillus niger *MTCC 2208 strains were streaked on three PDA slant test tubes incubated at 30°C for 7 days. All the media and chemicals utilized in this work were procured from HIMEDIA. After the growth period, 3 slant strains were serially diluted with distilled water (10^-1^ to 10^-7^).

*Preparation of growth media*

Four growth media were prepared with different concentrations of supplements such as D-Glucose, L-Asparagine and Thiamine and designated as ; Growth media I ( Potato Dextrose Broth 24 g/L, D-Glucose 40 g/L, L-Asparagine 2g/L, Thiamine 0.003 mg/L ) ; II (Potato Dextrose Broth 24 g/L, D-Glucose 60 g/L, L-Asparagine 4 g/L, Thiamine 0.005 mg/L), III ( Potato Dextrose Broth 24 g/L, D-Glucose 80 g/L, L-Asparagine 6 g/L, Thiamine 0.008 mg/L) and IV (Potato Dextrose Broth 24 g/L) respectively. The pH of all growth media were maintained at 5.1 ± 1.

*Inoculation*

Each 1 mL of 10^-7^ spores/mL dilution of *Aspergillus niger *MTCC strains were inoculated in all the four multivariate growth media in order that the three strains be exposed to be supplemented as well as non-supplemented media. All the flasks were incubated in orbital shaker incubator at 30°C with 180 rpm.

*Recovery*

Incubation of three different strains was terminated at 72 h, 96 h and 120 h. Mycelia were aseptically harvested by filtration through weighed silkscreen nylon filters and washed with distilled water until a clear filtrate was obtained and also were dried at 65°C to reach a constant weight.

*Extraction*

Aseptically 0.1 g dried mycelium was finely ground and transferred to weighed sterile centrifuge tubes. The content was suspended with 3 mL of 1M NaOH at room temperature and autoclaved. The sterile tubes were centrifuged at 12000 rpm for 15 min. The alkali insoluble fractions were separated and the supernatant was discarded. Residues were washed three times with distilled water and recentrifuged at neutral pH of 7. Residues were further extracted with 2% acetic acid (1:40 w/v) at 95°C for 8 h followed by centrifugation at 12000 rpm for 15 min. Insoluble fractions were discarded and pH was adjusted to 10 with 2M NaOH. Centrifugation was repeated and the precipitates were collected and washed with distilled water and weighed. Again, it was washed with ethanol (1:20 w/v) and then with acetone (1:20 w/v) (3). Precipitates of chitosan were dried at 60°C to a constant weight and tabulated in Tables 1, 2 and 3.

**Table 1:** Biomass (g/L) and Chitosan yield (%) of *A. niger *MTCC 872

**Media**	**Growth** **medium I**			**Growth** **medium II**			**Growth** **medium III**			**Growth medium IV**	
**Incubation periods (h)**	72	96	120	72	96	120	72	96	120	72
**Biomass (g/L)**	9.8	12.	13.3	14.4	15.2	16.2	15.9	19.0	19.8	3.2
**Chitosan Yield (%)**	17.2	19.3	19.1	18.3	20.2	21.2	21.1	22.0	23.1	9.1

**Table 2:** Biomass (g/L) and Chitosan yield (%) of *A. niger *MTCC 2208

**Media**	**Growth medium I**			**Growth medium II**			**Growth medium III**			**Growth medium IV**
**Incubation periods(h)**	72	96	120	72	96	120	72	96	120	72
**Biomass (g/L)**	12.0	13.3	14.2	16.3	17.4	18.5	17.9	19.3	20.8	4.2
**Chitosan Yield (%)**	21.2	21.4	22.0	23.4	23.2	24.2	25.1	25.1	26.1	10.0

**Table 3:** Biomass (g/L) and Chitosan yield (%) of *A. niger *MTCC 1785

**Media**	**Growth medium I**			**Growth medium II**			**Growth medium III**			**Growth medium IV**
**Incubation periods (h)**	72	96	120	72	96	120	72	96	120	72
**Biomass (g/L)**	8.41	9.97	11.8	12.1	13.2	15.5	14.2	15.9	17.3	2.8
**Chitosan Yield (%)**	18.3	19.0	20.0	20.0	21.2	22.2	23.2	23.1	25.2	9.3

*Evaluation*

The Maximum yield of fungal chitosan obtained from supplemented medium III was subjected to further evaluations. Physical appearance of obtained chitosan was observed; the Moisture content was determined by drying the sample to constant weight and measuring the sample after and before drying and the percentage was determined. The percentage of ash content was determined by the ratio between weight of residue and sample weight after being heated in muffle furnace and then cooled. The solubility was determined by using various solvents. The degree of deacetylation and infrared spectroscopy was calculated with standard methods (5-7). Infrared spectrum was recorded with a SHIMADZU Fourier transform infrared spectroscopy (FTIR) 8400S using a 100 mg KBr disk for the reference. The IR spectrum of produced fungal chitosan was compared with that one of commercial chitosan (HDV Chem Pharm, Chennai). The Antimicrobial susceptibility test was also evaluated.

**Table 4:** Comparison of anti microbial activity of produced chitosan and standard antibiotics

**Organisms**	**Chitosan Loaded Disc** **IZD in mm**	**Acetic Acid Loaded Disc** **IZD in mm**	**Ciprofloxacin Discs (30μg)** **IZD in mm**
Salmonella typhi	26	22	28
Salmonella paratyphi-A	30	26	23
Escherichia coli	20	16	14
Proteus vulgaris	25	20	10
Pseudomonas aeruginosa	30	28	30

*Anti-microbial susceptibility test*

Antimicrobial susceptibility was done for Clinical isolates, *Salmonella typhi, Salmonella paratyphi-A, Escherichia coli, Proteus vulgaris, *and *Pseudomonas aeruginosa *obtained from Bose lab, Madurai using disc diffusion method (8, 9).

## Results and Discussion

The Previous study reveals that late exponential phase produced the most extractable chitosan (3). All the three strain produced good mass of mycelia particularly with supplemented medium (I, II, III) rather than non-supplemented medium (IV). The maximum biomass for the three fungal strains *Aspergillus niger *MTCC 872 (19.8 g/L), *Aspergillus niger *MTCC 2208 (20.8 g/L) and Aspergillus *niger *MTCC 1785 (17.3 g/L) was seen in the runs performed with the Growth Medium III at 120 h. Chitosan production may be influenced mostly by L-Asparagine followed by D-Glucose and thiamine. The media were designed to obtain the maximum yield. No interaction effects among these three factors were reported (10). The maximum yield of chitosan in 120 h supplemented culture of *Aspergillus niger *MTCC 872 was 25.2%. The chitosan yields at the same incubation period on three concentrations supplemented cultures which were 20.0%, 22.2% and 25.2%. The maximum yield of chitosan in 120 h supplemented culture of *Aspergillus niger *MTCC 1785 was 23.1%. The chitosan yields of the same organisms at the same incubation period on three concentrations supplemented cultures which were 19.1%, 21.2% and 23.1%. The maximum yield of chitosan in 120 h supplemented culture of *Aspergillus niger *MTCC 2208 was 26.1%. The chitosan yields of the same organisms at the same incubation period on three concentrations supplemented cultures which were 22.0%, 24.2% and 26.1% and in which, non-supplemented 120 h culture of *Aspergillus niger *MTCC 2208 was 10%. Comparatively from the results highest yield (26.1%) was obtained, run proceeded containing PDB with maximum supplements in maximum incubation (Table 2).

 However, the yield was quite close to 72 and 96 h (25.1%). Since the three runs differ only in their cultivation time, the 72 h should be preferred in practical terms. Results from traditional chitin sources like mollusc and crustacean shells have been widely reported (11, 12). No reports were seen relating to microbial chitosan production in industries. In order to sustain the marine biodiversities, it is advisable to use micro-organism for the chitosan production in industry. However, recent advances in fermentation technology suggest that large-scale culturing of an organism, containing chitin and deacetylated chitin, might be an attractive route for producing this polymer. Several investigation have indeed shown that some strains of Mucor (13, 14), contain significant quantities of chitin, the maximum of which was extracted (8.71 g/L) using supplemented medium. The physical appearance of the produced chitosan was pale, white and flaky and its moisture content was 10.9%. Different types of chitosan were available in market such as: high molecular weight chitosan, low molecular weight chitosan, water-soluble chitosan, pharm grade chitosan, high density chitosan and chitosan oligosaccharide. These types of chitosan have different solubility. In our study, the produced fungal chitosan was insoluble in water and alkali solutions but soluble in organic acid (99.8% in 5% acetic acid) and partly in inorganic acids (diluted hydrochloric acid and nitric acid). The ash content percentage of produced fungal chitosan was found to be 0.89%. The degree of deacetylation was 85%. The IR spectrum of produced fungal chitosan was similar to that of the commercial chitosan, which indicates a strong similarity of the both compounds. Macro porous artificial skin containing antibiotics was prepared by lyophilization of chitosan/polyvinyl alcohol (PVA) blendmer, which could protect the wound surfaces from bacterial invasion by suppressing bacterial proliferation effectively (15). The diameter of the inhibitions zone of produced fungal chitosan loaded with acetic acid on *Salmonella typhi *(26 mm)*, S. paratyphi-A *(30 mm)*, E. coli *(20 mm)*, Proteus vulgaris *(25 mm) and *Pseudomonas aeruginosa *(30 mm), confirmed that the produced fungal chitosan has minimal antimicrobial activity.

## Conclusion

Finally, we can conclude that the strains used in the above work are promising sources of chitin. By recent developments in pharmaceutical biotechnology, these strains can be genetically manipulated and further optimised for getting better results and can also be adapted by industries in producing a quality and economical chitosan as an alternative to the shell fish derived product.

## References

[1] Brugnerotto J, Lizardi J, Goycoolea F M, Arguelles-Monal W, Desbrieres J, Rinaudo M (2001). An infrared investigation in relation with chitin and chitosan characterization. Polymer.

[2] Patel JK, Patel RP, Amin AF, Patel MM (2005). Formulation and evaluation of mucoadhesion glipizide microsphere. AAPS PharmSciTech.

[3] Pochanavanich P, Suntornsuk W (2002). Fungal chitosan production and its characterization. Lett. Appl. Microbiol.

[4] Attama AA, Onuigbo BE (2008). Preparation of stabilized mucoadhesive microparticles containing ciprofloxacin for eradication of Enterobacteriaceae. J. Pharm. Res.

[5] Amorim RVS, Souza W, Fukushima K, Campos-Takaki GM (2001). Faster chitosan production by mucoralean strains in submerged culture. Braz. J. Microbiol.

[6] Mortazavi SA (2004). Evaluation of the efficacy of several phytopolymers in terms of their duration of mucoadhesion. Iranian J. Pharm. Res.

[7] Khalaf SA (2004). Production and characterization of fungal chitosan under solid-state fermentation conditions. Int. J. Agri. Biol.

[8] Department of Health, Scottish Home and Health Department Welsh (1993). British Pharmacopoeia.

[9] Ministry of Health, Family Welfare (1985). Pharmacopoeia of India.

[10] Takaki (2003). Effect of medium components and time of cultivation on chitin production by Mucor circinelloides (Mucor javanicus IFO 4570) - A factorial study. Rev Iberoam Micol.

[11] Austin RR, Brine CJ, Castle JE, Zikakis JP (1981). Chitin: new facets of research. Science.

[12] Shepherd R, Reader S, Falshaw A (1997). Chitosan functional properties. Glycoconjugate J.

[13] Bartnicki-Garcia S, Nickerson WJ (1962). Isolation, composition and structure of cells walls of filamentous and yeast-like forms of Mucor rouxii. Biochem. Biophys. Acta.

[14] White SA, Farina PR, Fulton I (1979). Production and isolation of chitosan from Mucor rouxii. Appl. Environ. Microbiol.

[15] Adav AYY, Hise SBB (2008). Scientific correspondence, chitosan: a potential biomaterial effective against typhoid. Curr. Sci.

